# Supplementary material for: Does tinnitus and emotional distress influence central auditory processing? A comparison of acute and chronic tinnitus in normal-hearing individuals
Source: PLoS One. 2025 Jul 28;20(7):e0327777. doi: 10.1371/journal.pone.0327777 (PMC12303274; doi:10.1371/journal.pone.0327777)
Supplement: Attachment — Submitted filename: Response to Reviewers.docx [file pone.0327777.s003.docx]

**Dear Dr. Hantong Hu,**

Thank you for the opportunity to revise our manuscript “**Does Tinnitus and Emotional Distress Influence Central Auditory Processing? A Comparison of Acute and Chronic Tinnitus in Normal-Hearing Individuals**.” We are grateful to the editor and reviewers for their thorough evaluation and constructive feedback. We have carefully addressed each comment and revised the manuscript accordingly. Below, we provide a point-by-point response to the Editor’s requirements and to Reviewer #1, Reviewer #2, and Reviewer #3. Changes in the manuscript are marked, with original and revised text provided along with line numbers for clarity.

**Editor’s Comments and Required Revisions**

**Editor Comment 1**: ***“Please ensure that your manuscript meets PLOS ONE's style requirements, including those for file naming. The PLOS ONE style templates can be found at …”***

**Response:** We have revised the manuscript to conform to PLOS ONE’s formatting and style guidelines. This includes proper file naming and formatting of headings, figures, tables, and references as per the PLOS templates. All sections have been reviewed for compliance with PLOS style.

**Editor Comment 2: *“Please provide additional details regarding participant consent. In the ethics statement in the Methods and online submission, specify (1) whether consent was informed, and (2) what type was obtained (written or verbal, and if verbal, how documented and witnessed). If minors were included, state if parental consent was obtained. If consent was waived, note that.”***

**Response:** Thanks for your insightful feedback. We have clarified the Ethics Statement in the Methods to explicitly state the type of consent and that it was informed. In our study, all participants were adults and provided written informed consent (no minors were included, so parental consent was not applicable). The Ethics Statement now reads:

- ***Original:*** *“This study was approved by the Ethics Committee of the Shanghai Ninth People’s Hospital (Approval number: SH9H-2-22-T379-1), located in Shanghai, China. Written consent was obtained for each participant.”*
- ***Revised:*** *“This study was approved by the Ethics Committee of the Shanghai Ninth People’s Hospital (Approval number: SH9H-2-22-T379-1).* *All participants were adults aged 18 years or older, and written informed consent was obtained from each participant* *prior to inclusion in the study.”*

**Editor Comment 3:** ***“Thank you for stating in your Funding Statement: ‘This work was supported by the Shanghai Science and Technology Commission [Grant number 22Y11902000]; … Ninth People's Hospital Horizontal Project [Grant number JY2023-004].’ Please provide an amended statement that declares all funding or sources of support (external or internal) received during this study, as detailed in our guide for authors. Please also include the statement ‘There was no additional external funding received for this study.’ in your updated Funding Statement.”***

**Response:** We have amended the Funding Statement to list all sources of support and to include the required declaration of no additional funding. The revised Funding Statement now explicitly names the funding sources and adds that no other external funding was received. This update will be included in our cover letter and the manuscript. Specifically:

- ***Original Funding Statement:*** *“This work was supported by the Shanghai Science and Technology Commission [Grant number 22Y11902000]; the Shanghai Jiao Tong University Medical College Affiliated Ninth People's Hospital Horizontal Project [Grant number JY2023-004].”*
- ***Revised Funding Statement:*** *“This work was supported by the Shanghai Science and Technology Commission (Grant No. 22Y11902000) and the Shanghai Jiao Tong University Medical College Affiliated Ninth People's Hospital Horizontal Project (Grant No. JY2023-004).* ***There was no additional external funding received for this study.****”*

All sources of support are now disclosed, and the statement of no other funding has been added as requested.

**Editor Comment 4: *“In the online submission form, you indicated that ‘the data supporting this study’s findings are not available to the public due to participants’ confidentiality but are available from the corresponding authors on request.’ PLOS data policy requires all data underlying the findings to be fully available without restriction (with rare exception). If data cannot be made public for ethical or legal reasons, please explain and request an exemption.”***

**Response:** We have revised the Data Availability Statement to comply with PLOS ONE’s data policy. All data underlying our findings have now been made freely available in anonymized form. Specifically, we have prepared a de-identified dataset as Supporting Information and indicated this in the manuscript. The Data Availability section of our revised manuscript now states that *“All relevant data are within the manuscript and its Supporting Information files.”* If necessary, we will also deposit the data in a public repository and provide the DOI. We have removed the previous restriction (“available on request”) by anonymizing sensitive information, thereby addressing the confidentiality concern. In summary, all data are now publicly available either in the supplementary files or via a public repository, in accordance with PLOS policy.

**Editor Comment 5:** *“PLOS requires an ORCID iD for the corresponding author. Please ensure you have an ORCID iD and that it is validated in Editorial Manager (by clicking the Fetch/Validate link next to the ORCID field).”*

**Response:** We have verified that the corresponding author has an ORCID iD and it is validated in the Editorial Manager system. The ORCID iD has been linked and authenticated as required.

**Editor Comment 6:** *“Please include captions for your Supporting Information files at the end of your manuscript, and update any in-text citations to match. See our Supporting Information guidelines for more information.”*

***Response:*** We have added a section at the end of the manuscript listing captions for all Supporting Information files. Each supporting figure or table (e.g., any supplementary audiograms or data files) now has a caption (labeled as S1, S2, etc.) included after the References section. We also cross-checked and updated any in-text references to these supplementary items to ensure they are correctly labeled (e.g., “S1 Table”, “S1 Figure”). This addition ensures compliance with PLOS ONE’s guidelines on Supporting Information. we have created a minimal data supplement to fulfill data sharing requirements as noted above.

**Reviewer #1:**

**Comment (Reviewer #1):** *“The manuscript is well written with appropriate language. The title is focused, the abstract concise and clear. The introduction covers the background. Methods, results, and statistics are appropriate and clearly interpreted. The discussion, though long, is logical and links findings to existing literature. Significance and limitations are well stated. References are recent and well organized. Tables and figures are clear. The conclusion is short, clear, and well stated. Overall, this is a relevant manuscript with well-conducted experiments and valuable clinical implications. The findings (emotional distress in acute phase exacerbating difficulties) highlight the importance of early psychological support – an important take-home message for clinicians. Therefore, this manuscript can be considered appropriate and fit for publication.”*

**Response:** We sincerely thank Reviewer #1 for the positive and encouraging assessment of our work. We are pleased that the reviewer found the manuscript well-written, clear, and clinically relevant. We have taken note of the reviewer’s remarks regarding the length of the Discussion and the clarity of the Conclusions. In line with suggestions from the other reviewers, we streamlined the Discussion to remove redundancies and combined the concluding remarks into a single Conclusion section to improve clarity (see our responses to Reviewer #3 for details). We also performed minor edits for clarity and consistency throughout the manuscript. These changes further improve readability while preserving the content that Reviewer #1 praised. We appreciate Reviewer #1’s supportive comments and have maintained the strengths noted (focused title, clear abstract, thorough introduction, etc.) in the revised manuscript. Once again, thank you for your positive feedback and for highlighting the key message about early psychological support, which we have ensured remains prominent in the revised Conclusion.

**Reviewer #2:**

**Comment (Reviewer #2): **“Dear authors: Congratulations on your work. You have effectively connected tinnitus, central auditory dysfunction, and emotional distress, which is an important area of research. However, I believe there are opportunities to further strengthen the scientific impact of your study. While the connections made are solid, the work could benefit from more scientific exclusivity and disruptive hypotheses. (Not your purpose here but as an idea, why not integrate new technologies and more precise emotional assessment tools?)***

***The Materials and Methods section demonstrated technical rigor in auditory testing in a well-controlled environment. However, the study would have been more robust with a larger sample size, longitudinal follow-up, and a more comprehensive emotional assessment as said above. I also feel that neurofunctional measures, although not your original purpose, could have further enriched the study – this could be an excellent direction for future research.***

***Acute and chronic tinnitus patients showed significant deficits in central auditory processing (elevated GDT thresholds, poorer speech-in-noise, reduced TEOAE suppression), which validates central auditory impairment. However, the minimal differences between acute and chronic phases could have been better explored using advanced neuroimaging methods (EEG or fMRI), as well as big data approaches to track neuroplastic changes over time. Additionally, the correlational analyses would have benefitted from multivariate control to account for other influencing factors.***

***The correlation between emotional scores (THI and VAS) and speech-in-noise performance is important, yet reliance on THI and VAS alone limits psychological insight. Incorporating validated scales for anxiety and depression, alongside advanced techniques like machine learning models to predict clinical evolution, could offer a more nuanced emotional analysis.***

***To conclude, your study provides valuable descriptive evidence regarding the impact of tinnitus on central auditory processing, but it remains largely descriptive with very limited innovation. It would benefit from deeper exploration through neuroimaging and more comprehensive emotional assessments... Once again, congratulations on the work, and I look forward to seeing how future studies in this area evolve.”****

**Response:** We appreciate your positive appraisal of our methodological rigour and your thoughtful suggestions for strengthening the scientific reach of our work. Although the present study was designed as a behavioral comparison, we fully agree that advanced neuro-imaging, richer emotion scales, and multivariate or machine-learning analyses would deepen future investigations. The following changes on Limitation have been made:

**Revised：**

***“Limitations.*** This single-centre study enrolled a modest sample (n = 30 per tinnitus group) of adults aged 18–55 years with normal audiograms; a larger, multi-site cohort spanning younger and older patients—including those with concomitant hearing loss—will be needed to confirm the present findings. Because the design was cross-sectional, we could not follow neuroplastic changes as tinnitus progresses; prospective longitudinal studies coupled with EEG or fMRI are required to chart that trajectory. Emotional distress was assessed only with THI and VAS; adding validated anxiety- and depression scales, and analysing data with multivariate or machine-learning methods, would provide a fuller account of the psychological factors that interact with auditory function.*”*

These additions directly reflect your recommendations while keeping the manuscript focused and concise. Thank you again for your constructive guidance.

**Reviewer #3:**

**General Comment 1 (Objectives and Study Design): *“The main issue is that the objectives, hypothesis, and study design are not clearly described. The introduction ends with a rather vague statement about the goals of the study, leaving many open questions regarding the study design. There should be a dedicated paragraph at the end of the introduction clearly describing why the different measures were used and what the specific research question of this study was. This needs to be more clearly described. Please modify.”***

**Response:** We agree with the reviewer that the introduction should more clearly articulate the specific objectives and rationale of our study, including why we chose measures. We have added a dedicated concluding paragraph to the Introduction to explicitly state our research questions and the study design. In the revised Introduction, we outline the purpose of the study, the key auditory measures used, and the hypotheses regarding acute vs. chronic tinnitus. Specifically, we now explain that we used the gap detection test (GDT), the Mandarin Hearing in Noise Test (MHINT), and contralateral suppression of TEOAEs to assess different aspects of central auditory processing (temporal resolution, speech-in-noise recognition, and efferent function, respectively) in acute and chronic tinnitus patients versus controls. We also clearly state the hypothesis that emotional distress may influence these auditory processing outcomes. This added paragraph (last paragraph of Introduction) provides a focused summary of the study’s aims and design, as requested.

- **Original (Lines 104–112):** *“To our knowledge, this is the first study to investigate differences in central auditory processing function between patients with acute and chronic tinnitus. The purpose of this study is to evaluate whether tinnitus affects central auditory function in individuals with normal hearing and to explore the role of emotional distress and neural plasticity in this process. If functional changes are present, this study seeks to determine whether these changes are stable or influenced by dynamic processes over time. Understanding these mechanisms will provide valuable insights into tinnitus and offer important clinical implications for diagnosing and managing tinnitus in individuals with normal hearing.”*
- **Revised:** *“To our knowledge, this is the first study to investigate differences in central auditory processing function between patients with acute and chronic tinnitus.* *We employed the gap detection test (GDT), Mandarin Hearing in Noise Test (MHINT), and contralateral suppression of TEOAEs to assess temporal resolution, speech-in-noise recognition, and efferent auditory function, respectively. The purpose of this study is to evaluate whether tinnitus affects central auditory function in normal-hearing individuals and to explore* *how emotional distress and neural plasticity* *may contribute to any observed changes. If functional changes are present, we further seek to determine whether these changes* *differ between acute and chronic stages of tinnitus or remain stable over time. Understanding these mechanisms will provide valuable insights into how tinnitus* *evolves and offer important clinical implications for managing tinnitus.”*

This revised Introduction now clearly identifies what we are testing and why, directly addressing the reviewer’s concern.

**General Comment 2 (Group Definitions – Acute vs. Chronic Tinnitus): *“The second main issue is that the group definitions are not clear.*** ***How was acute and chronic tinnitus differentiated? Why was this distinction made in the first place? How is it defined? How was the presence of tinnitus clinically assessed, and how were the participants recruited? Why was the cut-off of 3 months chosen? Please explain in more detail. It would also be important here to provide approximate information on how long the chronic tinnitus patients experienced tinnitus.”***

**Response:** We appreciate the opportunity to clarify our group definitions and recruitment methods. We now define **acute tinnitus** as < 3 months and **chronic tinnitus** as ≥ 3 months in *Methods*. The 3-month cut-off follows clinical practice (Japanese Guideline 2020; German Guideline 2022) and recent research (Zhang et al., 2023). We have added this explanation to the manuscript to justify the distinction.

We also now describe how tinnitus presence was assessed and how participants were recruited. All tinnitus patients were initially evaluated by an otolaryngologist and underwent audiological examination (to confirm normal hearing) as well as tinnitus pitch and loudness matching. We recruited tinnitus participants from the Otolaryngology clinic of our hospital (Shanghai Ninth People’s Hospital) during the study period. The presence of subjective tinnitus was confirmed clinically through patient history and tinnitus matching. The control group consisted of healthy volunteers with normal hearing and no history of tinnitus, recruited via hospital staff and community advertisement, age-matched to the patient groups. We have explicitly stated that control participants were screened to ensure they did not have tinnitus (even minor or transient tinnitus episodes were grounds for exclusion from the control group). This screening was done by directly questioning controls about any perception of ringing in their ears. We added a sentence in the Methods: “Control participants were recruited via advertisement and interviewed to ensure they had no perceived tinnitus and thus did not complete tinnitus-related questionnaires.”

Regarding why the distinction was made: Our rationale was that comparing acute and chronic tinnitus might reveal whether central auditory processing changes worsen over time or stabilize (i.e., to test if chronic tinnitus patients show greater deficits than acute patients, or if neuroplastic adaptation occurs). We have clarified this rationale in Introduction. In the Introduction, we now state that comparing these groups can “reveal critical differences and provide insights into how tinnitus evolves”.

Additionally, we added the mean chronic-tinnitus duration (~2.6 years, range 4 months–10 years) to the Participants section for context.

We added this detail to the Participants section for context.

- **Original:** *“The study involved 30 individuals with acute subjective tinnitus (lasting more than 3 days but under 3 months), 30 with chronic subjective tinnitus (lasting more than 3 months), and 30 healthy volunteers as controls.”*
- **Revised:** *“The study involved 30 individuals with acute subjective tinnitus (**duration <3 months), 30 with chronic subjective tinnitus (**duration >3 months), and 30 healthy volunteers as controls.* *We selected a 3-month cutoff based on clinical guideline to distinguish acute (early-stage) tinnitus from chronic tinnitus*(22)*. All participants had normal hearing as defined below. Participants with tinnitus were recruited from our hospital’s otolaryngology clinic, and each had their tinnitus diagnosis confirmed via clinical assessment (including history and* *tinnitus matching). Chronic tinnitus patients in our sample had experienced tinnitus for a mean duration of approximately 2.6 years (ranging from ~4 months to 10 years). Control volunteers were age-matched individuals with normal hearing and no history or complaint of tinnitus.* *Control participants were recruited via advertisement and interviewed to ensure they had no perceived tinnitus and thus did not complete tinnitus-related questionnaires.”*

Ogawa K, Sato H, Takahashi M, Wada T, Naito Y, Kawase T, et al. Clinical practice guidelines for diagnosis and treatment of chronic tinnitus in Japan. Auris Nasus Larynx. 2020;47(1):1-6. Epub 2019/10/14. doi: 10.1016/j.anl.2019.09.007. PubMed PMID: 31606294.

**General Comment 3 (Role of Control Group):** ***“Another issue is that there was a control group included; however, in the presentation of the results and the tables, often the results for the control group are not shown. Therefore, it is not fully clear why the control group was included. This is not really clear throughout the study. It is not clear what the control group is for, as these were not included as its discussion is often incoherent. Please improve this and explain.”***

**Response:** We apologize for any lack of clarity regarding the role of the control group. The control group of normal-hearing individuals without tinnitus was included to provide a baseline for comparison – to determine how tinnitus patients (acute or chronic) differ from individuals with no tinnitus on central auditory processing tasks. We have made this purpose explicit in the Introduction and Methods, and we have ensured that control data are appropriately presented in the Results and discussed in the Discussion.

In the Results section, we have double-checked that control group results are included wherever relevant. Indeed, our statistical analyses for GDT, MHINT, and TEOAE suppression were designed to compare all three groups (Acute T, Chronic T, Control). For example, we report that both the acute (AT) and chronic (CT) tinnitus groups had significantly worse performance than controls on certain measures, with no significant difference between AT and CT – highlighting that the control group establishes the normal performance level. We have edited the text to explicitly mention the control group in the description of results, so their inclusion is clear. For instance, in Section 3.2 we now say: “Post hoc t-tests with Bonferroni correction showed that both acute and chronic tinnitus groups had significantly lower CS compared to controls (*p* < 0.05), but no significant difference between the acute group and chronic group (*p* > 0.05).”. Similarly, for MHINT SRT and TEOAE suppression, we explicitly mention comparisons to control.

We also noticed that Table 1 (Participant characteristics) in the original submission did not list explicit audiological values for the control group, which may have caused confusion. We have updated Table 1 to include the pure-tone audiometry results for all groups (e.g., average hearing thresholds), confirming that controls indeed had normal hearing comparable to the tinnitus groups (with no significant differences in hearing thresholds). Additionally, we clarified in the legends and text which tables/figures include control data. For example, Figure 1A and 1B include control comparisons (and are described as such), Figure 2 (GDT results) includes control group data, etc. We corrected any oversights where control data or labels might have been omitted.

In the Discussion, we now explicitly discuss the control group’s role. We emphasize that tinnitus patients showed central processing deficits relative to controls, and that the absence of differences between acute and chronic tinnitus (with both differing from controls) suggests that the central auditory impact of tinnitus is already present in the acute stage and does not markedly progress. By highlighting the contrast with the control group, we underscore why the control was necessary: to establish that tinnitus (regardless of duration) impairs certain functions beyond normal variation.

To avoid any incoherence, we made sure every mention of results clearly indicates the group being referred to (AT, CT, or Control). For example, instead of saying “both groups,” we specify “both tinnitus groups relative to controls.” We also removed any redundant or confusing sentences that might have obscured the control comparisons.

Summary of changes regarding the control group: We included control group results in all relevant tables and figures, clarified the description of those results in the text, and stated the purpose of the control group in the Introduction/Methods (as a baseline for normal performance). We also added a note in Methods (Participants) that control subjects did not undergo THI/VAS since they have no tinnitus (as mentioned earlier), to avoid any confusion about “missing” data for controls. The control group’s role should now be evident throughout the manuscript.

**General Comment 4 (Discussion Length and Redundancy):** ***“A fourth issue is that the discussion section is quite long and in many parts redundant, with redundant description of the conclusions (twice) and an additional significance statement, which should be condensed and combined into one paragraph or integrated in the general discussion. Otherwise the discussion is confusing for the reader.”***

**Response:** Response: Thank you for this valuable feedback. We agree that the Discussion was overly long and contained redundant elements. In response, we have carefully revised this section to enhance clarity and flow. Specifically:

- We removed overlapping content by eliminating the separate “Significance” paragraph and merging the two previously distinct conclusion sections into a single, cohesive final paragraph.
- We reorganized the Discussion to ensure each finding is discussed once and in a logical sequence, avoiding repetition across paragraphs.
- The final Conclusion paragraph now clearly summarizes the key findings and clinical implications without reiterating points made earlier in the Discussion.

These revisions have shortened the Discussion, reduced redundancy, and improved readability, in line with the reviewer’s suggestion.

**General Comment 5 (Excessive Use of Abbreviations): *“A style aspect is that too many abbreviations are used which only makes the text hard to read. I do not think it is necessary to abbreviate acute tinnitus (AT) and chronic tinnitus (CT). Also the abbreviation for the control group as CL is not intuitive. Try to reduce the use of abbreviations throughout the text; the reader will thank you for that.”***

**Response:** We appreciate this stylistic suggestion. We have reduced the use of abbreviations to improve readability. In particular:

- We removed the abbreviations “AT”, “CT”, and “CL” for acute tinnitus, chronic tinnitus, and control group, respectively. Throughout the text, we now generally write these terms in full (e.g., “acute tinnitus group” or “chronic tinnitus group” and “control group”) to avoid overloading the reader with acronyms. We retained abbreviations only where they are standard and widely understood (e.g., THI, VAS, GDT, MHINT, TEOAE, which are common audiology terms), but even for these, we ensured each is defined once and used judiciously.
- In places where abbreviations were used excessively, we replaced them with plain language. For example, in the Results and Discussion, instead of “AT vs. CT”, we now say “acute vs. chronic tinnitus groups”. In table headings and figure captions, we also revised labels to be clearer (e.g., “Acute Tinnitus” and “Chronic Tinnitus” spelled out).
- We eliminated the abbreviation “CL” for the control group; we simply use “Controls” or “Control group” now.

We have proofread the manuscript to ensure all unnecessary abbreviations have been expanded. We believe this addresses the reviewer’s concern and indeed makes the manuscript easier to read.

**General Comment 6 (Data Availability Statement): *“Please include a statement on data availability.”***

**Response:** We apologize for the omission. As also required by the editor (Editor Comment 4), we have now included a Data Availability Statement in the manuscript. It appears after the References section, as per PLOS ONE format. The statement explicitly indicates how the data can be accessed. As noted earlier, we have made all relevant data available either within the manuscript or as Supporting Information. For example, the Data Availability Statement in the revised manuscript reads: *“Data Availability Statement: All relevant data are within the manuscript and its Supporting Information files.”*

If needed, we will update this to a public repository link, but the key point is that the statement is now present and compliant. This addresses the reviewer’s request.

In addition to the general comments above, Reviewer #3 provided **specific comments** referring to line numbers in the original manuscript. We address each of these in detail below:

**Specific Comment (Line 80):** ***“It is not clear how the topic of neuroplasticity relates to the previous paragraphs. It might be one of multiple reasons for tinnitus. Here you state that tinnitus is an adaptive response due to reduced peripheral input. This hypothesis needs to be elaborated and explained in more detail.”***

**Response:** We have expanded the discussion of neuroplasticity in the Introduction to clearly link it with the prior context and to elaborate on its role in tinnitus. Originally, we introduced neuroplasticity somewhat abruptly. We now provide a smoother transition and more explanation: we explain that tinnitus may involve *maladaptive neuroplastic changes* in the central auditory system as the brain’s response to reduced peripheral input (e.g., hearing loss or damage). We also clarify that neuroplasticity is just one aspect of tinnitus pathophysiology. To support this, we added a reference to Shore *et al.*, 2016 (Nat Rev Neurol), which reviews how maladaptive plasticity in auditory pathways can trigger tinnitus.

- **Original:** *“Neuroplasticity refers to the structural or functional changes in the brain or nervous system in response to injury or stimulation. In tinnitus patients, neuroplasticity often manifests as an adaptive response within the central auditory system due to reduced peripheral auditory input, leading to the reorganization of neuronal activity (15).”*
- **Revised:** *“**Neuroplasticity refers to the structural or functional changes in the brain or nervous system in response to injury or stimulation. A leading model of tinnitus attributes the phantom percept to maladaptive plasticity within central auditory pathways that compensate for reduced peripheral input by “filling in” missing sound, thereby generating a persistent internal signal(15). When peripheral input is diminished, these central circuits up-regulate neural gain or reorganize frequency maps, creating a self-sustaining tinnitus percept.”*

Shore SE, Roberts LE, Langguth B. Maladaptive plasticity in tinnitus--triggers, mechanisms and treatment. Nat Rev Neurol. 2016;12(3):150-60. Epub 20160212.

**Specific Comment (Line 87):** ***“It is not fully clear why emotions should affect neuroplasticity? Why would emotional distress accelerate the process? What is the biological basis for this? How does it relate to neuroplasticity? This should be explained clearly and supported with adequate references. The hypothesis is not well motivated and the rationale for this is not clear. Please explain.”***

**Response:** Thank you for highlighting the need to clarify the link between emotional distress and neuroplasticity. We have expanded the Introduction to provide a stronger, evidence-based rationale:

**Revised:** “*Neuroplasticity refers to the structural or functional changes in the brain or nervous system in response to injury or stimulation.* *A leading model of tinnitus attributes the phantom percept to maladaptive plasticity within central auditory pathways that compensate for reduced peripheral input by “filling in” missing sound, thereby generating a persistent internal signal (15).* *When peripheral input is diminished, these central circuits up-regulate neural gain or reorganize frequency maps, creating a self-sustaining tinnitus percept. This maladaptive plasticity does not act in isolation: auditory regions is tightly interconnected with limbic structures such as the amygdala, hippocampus and anterior cingulate cortex; neuro-imaging shows that tinnitus distress is accompanied by enhanced amygdala-to-auditory-cortex connectivity (Chen et al., 2017)* *and broader limbic-auditory coupling (Singh et al., 2023).* *Emotion can interfere with cognitive functions such as attention and working memory. These findings provide a biological basis for emotional modulation of auditory plasticity: stress and anxiety heighten attention to tinnitus, repeatedly activating and reinforcing the maladaptive loop, whereas effective emotion regulation may allow the system to recalibrate.* *Accordingly, we hypothesize that* *emotional distress can accelerate tinnitus-related neuroplastic changes, potentially creating a vicious cycle, while alleviating the emotional burden might slow or even reverse this process*”.

Singh A, Smith PF, Zheng Y. Targeting the Limbic System: Insights into Its Involvement in Tinnitus. Int J Mol Sci. 2023;24(12). Epub 20230608. doi: 10.3390/ijms24129889. PubMed PMID: 37373034; PubMed Central PMCID: PMCPMC10298522.

Chen YC, Xia W, Chen H, Feng Y, Xu JJ, Gu JP, et al. Tinnitus distress is linked to enhanced resting-state functional connectivity from the limbic system to the auditory cortex. Hum Brain Mapp. 2017;38(5):2384-97. Epub 20170123. doi: 10.1002/hbm.23525. PubMed PMID: 28112466; PubMed Central PMCID: PMCPMC6866871.

**Specific Comment (Line 98):** ***“Why does chronic tinnitus lead to significant neural plasticity changes and acute does not? This sentence is not clear. What are the correlates for these neural plasticity changes? What exactly is changed? Please specify.”***

**Response:** Thank you for pointing out the need for greater specificity. We have revised the passage to delineate the distinct neural correlates observed in chronic versus acute tinnitus. We have clarified the sentence as follows:

*“Acute tinnitus patients typically exhibit fewer signs of outer hair cell damage, as indicated by distortion product otoacoustic emissions (DPOAE) recordings, compared to chronic tinnitus patients (10).* *In contrast, chronic tinnitus is associated with sustained neuroplastic reorganization—including stronger limbic–auditory connectivity (parahippocampal gyrus, amygdala), cortical hyperactivity, and altered attention networks (21–23).* *Clinically, acute tinnitus is often accompanied by anxiety, heightened tinnitus awareness, and poor sleep, while chronic tinnitus is more frequently linked to depressive symptoms (20).”*

Lan L, Li J, Chen Y, Chen W, Li W, Zhao F, et al. Alterations of brain activity and functional connectivity in transition from acute to chronic tinnitus. Hum Brain Mapp. 2021;42(2):485-94. Epub 20201013. doi: 10.1002/hbm.25238. PubMed PMID: 33090584; PubMed Central PMCID: PMCPMC7776005.

Lan L, Chen YC, Shang S, Lu L, Xu JJ, Yin X, et al. Topological features of limbic dysfunction in chronicity of tinnitus with intact hearing: New hypothesis for 'noise-cancellation' mechanism. Prog Neuropsychopharmacol Biol Psychiatry. 2022;113:110459. Epub 20211016. doi: 10.1016/j.pnpbp.2021.110459. PubMed PMID: 34666066.

Hu J, Xu JJ, Shang S, Chen H, Yin X, Qi J, et al. Cerebral Blood Flow Difference Between Acute and Chronic Tinnitus Perception: A Perfusion Functional Magnetic Resonance Imaging Study. Front Neurosci. 2021;15:752419. Epub 2021/10/23. doi: 10.3389/fnins.2021.752419. PubMed PMID: 34675772; PubMed Central PMCID: PMCPMC8523683.

**Specific Comment (Line 102): *“How is the transition from acute to chronic tinnitus defined? What is the exact clinical distinction? Please explain. Please also address the potential different causes for short acute tinnitus and chronic tinnitus.”***

**Response:** Thank you for requesting a clearer definition of the acute-to-chronic transition and the possible differences in etiology. We have updated the Introduction to include (i) the explicit 3-month clinical cut-off and (ii) typical precipitating factors for short- versus long-duration tinnitus. The revised paragraph now reads:

*“*Few studies have explored the complex relationship between emotions, neuroplasticity, and tinnitus (16, 19). Most clinical work has focused on chronic tinnitus, whereas acute tinnitus remains less studied (11, 14, 10, 17, 20, 21). Clinically, tinnitus persisting for < 3 months is considered acute, while tinnitus lasting ≥ 3 months is classified as chronic(Ogawa et al., 2020). Acute tinnitus is often linked to recent triggers such as noise trauma, ototoxic medication, or acute stress and may remit if the trigger resolves. By contrast, chronic tinnitus usually reflects more enduring cochlear damage and long-term neurophysiological change (Lan et al., 2021). Comparing acute and chronic cases therefore offers insight into how tinnitus evolves. Acute patients generally show less outer-hair-cell damage on DPOAE than chronic patients (10), whereas chronic tinnitus is associated with sustained neuroplastic reorganisation—including stronger limbic-auditory connectivity (parahippocampal gyrus, amygdala), cortical hyperactivity, and altered attention networks (21-23). Clinically, acute tinnitus tends to coincide with anxiety, heightened tinnitus awareness, and poor sleep, while chronic tinnitus is more often linked to depressive symptoms (20). Nonetheless, the precise neural changes that occur as tinnitus progresses—and how emotional distress modulates this trajectory—remain largely unknown*.*”

Ogawa K, Sato H, Takahashi M, Wada T, Naito Y, Kawase T, et al. Clinical practice guidelines for diagnosis and treatment of chronic tinnitus in Japan. Auris Nasus Larynx. 2020;47(1):1-6. Epub 2019/10/14. doi: 10.1016/j.anl.2019.09.007. PubMed PMID: 31606294.

Lan L, Chen YC, Shang S, Lu L, Xu JJ, Yin X, et al. Topological features of limbic dysfunction in chronicity of tinnitus with intact hearing: New hypothesis for 'noise-cancellation' mechanism. Prog Neuropsychopharmacol Biol Psychiatry. 2022;113:110459. Epub 20211016. doi: 10.1016/j.pnpbp.2021.110459. PubMed PMID: 34666066.

**Specific Comment (Line 107):** ***“How is the role of emotional distress and neural plasticity quantified here? The exact goal and objective of this study is not fully clear. This needs to be more clearly described. What is the objective and with which methods is this objective going to be addressed. This should be explained more clearly.”***

**Response:** Thank you for asking us to clarify our objectives and how emotional distress and putative neuroplastic changes are quantified. The final paragraph of the Introduction has been expanded accordingly:

*“To our knowledge, this is the first study to compare central auditory‐processing functions in patients with acute* *(< 3 months) and chronic (≥ 3 months) tinnitus. We administered* *three behavioural tests—**gap-detection Test (GDT) for temporal resolution, the Mandarin Hearing-in-Noise Test (MHINT) for speech-in-noise recognition, and contralateral suppression of transient evoked otoacoustic emissions (TEOAE) for efferent-pathway function—to normal-hearing participants.* *Emotional distress was quantified with the Tinnitus Handicap Inventory (THI) and a tinnitus loudness visual-analogue scale (VAS).* *By correlating THI/VAS scores with auditory outcomes, we infer how distress may modulate function; differences (or lack thereof) between acute and chronic groups are interpreted as behavioural markers of underlying neuroplastic adaptation.* *Our goals are therefore to* *(i) determine whether tinnitus per se alters central auditory processing, (ii) assess whether these alterations differ between acute and chronic stages, and (iii) examine the relationship between emotional distress and auditory performance. Understanding these links will provide valuable insights into how tinnitus evolves and offer important clinical implications for managing tinnitus.”*

This revision explicitly states the study’s objectives, maps each test to the auditory domain it assesses, and explains how emotional distress is quantified and related to putative neuroplastic changes.

**Specific Comment (Line 114):** ***“How were the participants recruited? What background did they have? Why were only young subjects recruited? Please explain in more detail.”***

**Response:** Thank you for requesting more detail on recruitment, participant background, and age range. We have expanded the Participants subsection to read:

*“The study, conducted from May 1, 2023, to May 1, 2024, involved 30 individuals with acute subjective tinnitus (duration <3 months; mean duration ≈ 0.6 months, range 3 days–2.5 months), 30 with chronic subjective tinnitus (duration >3 months; mean duration ≈ 2.6 years, range 4 months–10 years), and 30 healthy volunteers as controls (Table 1).* *We selected a 3-month cutoff based on clinical guideline to distinguish acute (early-stage) tinnitus from chronic tinnitus(22). All participants had normal hearing as defined below. Participants with tinnitus were recruited from our hospital’s otolaryngology clinic, and each had their tinnitus diagnosis confirmed via clinical assessment (including history and tinnitus matching). Chronic tinnitus patients in our sample had experienced tinnitus for a mean duration of approximately 2.6 years (ranging from ~4 months to 10 years). Control volunteers were age-matched individuals with normal hearing and no history or complaint of tinnitus. They were recruited by advertisement among hospital staff, relatives, and community members, and interviewed to ensure they had no perceived tinnitus; therefore, they did not complete THI or VAS questionnaires. All participants were aged between 18 and 55 years, as we restricted the age range to avoid the effects of presbycusis on the results.”*

These additions specify *how* participants were recruited, *why* we limited the cohort to younger adults, and the clinical criteria used to define acute versus chronic tinnitus.

***Specific Comment (Line 129): “How was normal hearing assessed? What were the results of the audiometry? Why are these not presented as a summary statistic for the groups? Please include.”***

**Response:** Thank you for pointing out the need to document our audiometric findings more explicitly. We have added the requested details:

- **Assessment procedure (Methods).** Pure-tone thresholds were measured at 0.25–8 kHz with a calibrated audiometer (Madsen Astera) and insert earphones, following ISO 7029 standards. Normal hearing was defined as ≤ 20 dB HL at all tested frequencies; tympanometry confirmed type-A tympanograms in every ear.
- **Summary statistics (Table 1).** A new row reports the mean pure-tone average (PTA, 0.25–8 kHz):Acute = 9.71$\pm$4.39 dB HL; Chronic = 8.54$\pm$3.61 dB HL; Control = 8.08$\pm$4.49 dB HL; One-way ANOVA: F (2, 87) = 1.405, p = 0.251.
- **Results text (Section 3.1).** We now state:
  *“All participants met the ≤ 20 dB HL criterion for normal hearing (see Table 1). Mean PTA did not differ significantly among acute, chronic, and control groups (F (2, 87) = 1.405, p = 0.251).”*

These additions clarify how normal hearing was established and make the group audiometric data readily accessible.

**Specific Comment (Line 172): *“SL or SPL?”***

**Response:** Thank you for noting this ambiguity. The contralateral white noise was presented at 50 dB SL (sensation level)—i.e., 50 dB above each participant’s detection threshold for that noise—rather than in HL or absolute SPL.

**Original*:*** *“...both with and without a 50 dB SL contralateral white noise suppressor delivered through an audiometer and insert earphones...”*

**Revised:** *“...both with and without a 50 dB SL contralateral white noise suppressor* *(i.e., 50 dB above each participant’s white noise threshold) delivered through an audiometer and insert earphones...”*

**Specific Comment (Line 193):** ***“dB SL? Do you mean SPL? Was a special MATLAB program used to perform the GDT task? Was it custom programmed? Were the stimuli presented via speakers or headphones? Was the gap ramped? Please include more information.”***

**Response:** Thank you for requesting additional methodological detail. Section 2.8 (Gap Detection Test) has been expanded as follows to address all of your questions:

*“The Gap Detection Test (GDT), measuring temporal resolution in milliseconds (ms), was assessed using a three-interval forced-choice procedure. We used a custom MATLAB (v7.0) program to present the stimuli via ER-3A insert earphones in a soundproof booth, at 40 dB SL (i.e., 40 dB above each participant’s threshold for the stimulus). Each trial included three 1000 ms narrow-band Gaussian noise bursts (**either centered at 1 kHz or 4 kHz; for tinnitus patients, one test used a noise band centered at their individual tinnitus frequency in lieu of 4 kHz).* *One of the three bursts contained a brief silent gap. The silent gap had an abrupt onset and offset (no ramping applied). The initial gap duration was 20 ms, and it decreased in 2-ms steps between trials until the participant failed to detect the gap. A two-down/one-up adaptive rule (1-ms step size) then refined the threshold. The participant’s task was to identify which burst had the gap, and the GDT threshold was defined as the shortest interval where participants consistently detected the gap.”*

These additions specify (i) that 40 dB refers to sensation level, not SPL; (ii) that a custom MATLAB program was used; (iii) that stimuli were presented via insert earphones, not loudspeakers; and (iv) that the gap edges were unramped. We trust this clarifies the procedure.

**Specific Comment (Line 214):** *“There are no audiological findings included in Table 1. Please check and explain. Also why were the psychological tests not performed in the control group as a verification? Please explain. Are the p-values reported in table the results of the ANOVA? Then more information about the ANOVA statistics should be reported? What test was used?”*

**Response:** Thank you for these important observations. We have revised Table 1 and the accompanying text as follows:

1. **Audiological data added.**
   *Table 1 now includes the mean pure-tone average (PTA, 0.25–8 kHz) for each group (Acute = 9.71*$\pm$*4.39 dB HL; Chronic = 8.54*$\pm$*3.61 dB HL; Control = 8.08*$\pm$*4.49 dB HL; One-way ANOVA: F (2, 87) = 1.405, p = 0.251.).*
2. **Why THI/VAS were not administered to controls.**
   THI and VAS quantify tinnitus severity; individuals in control group without tinnitus cannot provide meaningful scores. We now state explicitly in the Methods that control participants were screened to confirm the *absence* of tinnitus and therefore did not complete these questionnaires.
3. **Statistical notation clarified.**
   ***Table 1 footnote:*** **Note.** Age p value from one-way ANOVA (three groups). THI and VAS p values from independent-samples *t* tests (acute vs chronic only).

**In Results 3.1** we now report full statistics:

- Age: F(2,87) = 1.558, *p*=0.216
- PTA: F (2, 87) = 1.405, *p* = 0.251
- THI total: t(49) = 2.138, *p* = 0.038
- THI-Emotional: t(49) = 2.043, *p* = 0.047
- THI-Functional / Catastrophic and VAS: t(49) = 1.623, *p*=0.112; t(49) = 1.787, *p*=0.08; t(58) = 1.073, *p*=0.288, respectively

These additions ensure that audiological findings are transparent, clarify why psychological instruments were not applied to controls, and specify the statistical tests underlying every *p* value.

**Specific Comment (Line 218):** ***“The TEOAE results seem to be presented in Table 2. This is confusing. Please check which Tables show which information, and provide the correct information and description.”***

**Response:** We appreciate the reviewer’s observation. After a line‐by‐line check we found that the text at Line 218 erroneously cited “Table 2” for the overall TEOAE group comparison. The corrections are:

1. **Text citation.**
   *Line 218 now reads:*
   “Group differences among three groups in contralateral suppression (CS) of TEOAE are shown in Figure 1B. See **Table 2** for the ear‐specific values within the tinnitus groups.”
2. **Table captions and content.**
   *Table 2* “Contralateral suppression (CS) on TEOAEs for dominant and non-dominant ears in acute and chronic tinnitus groups”
   *Table 3* Spearman correlations among auditory measures and tinnitus scores
3. **Legends and cross-references.**
   All table and figure legends have been updated for clarity, and every in-text citation now points to the correct table or figure.

These revisions remove the ambiguity noted by the reviewer and ensure that TEOAE results are presented and referenced unambiguously throughout the manuscript.

**Specific Comment (Line 220):** ***“Please provide adequate reporting of ANOVA results, including F-statistic and degrees of freedom. Please do so also for the following results where this applies.”***

**Response:** Thanks for your suggestions. We have gone through the Results and added full ANOVA (and t-test) statistics wherever appropriate. All corresponding figure legends and Table 1 footnotes have been updated to indicate the test used. We hope this satisfies the requirement for complete and transparent statistical reporting.

**Specific Comment (Line 220 continued):** ***“Why were THI tests and VAS not tested in the control group? How was checked, whether the control group did not have tinnitus?”***

**Response:** We appreciate this query and have clarified the text accordingly:

- **Relevance of THI/VAS.** THI and VAS are diagnostic instruments intended only for individuals who perceive tinnitus. Administering them to participants who do not experience tinnitus would yield a score of 0 on every item and add no meaningful data.
- **Screening of control participants.** All prospective controls underwent a brief interview conducted by an audiologist. They were asked whether they had ever experienced persistent ringing, buzzing, or other phantom sounds in either ear. Only individuals who answered “no” to all tinnitus-related questions—and who had normal audiograms—were enrolled. Any candidate reporting current or recurrent tinnitus was excluded.

The revised sentence in the **Participants** subsection now reads:

“*They were recruited by advertisement among hospital staff, relatives, and community members, and interviewed to ensure they had no perceived tinnitus; therefore, they did not complete THI or VAS questionnaires.”*

**Specific Comment (Line 229): *“What statistical test was used to compare these results? Please explain and include this information.”***

**Response:** Thank you for requesting clarification of the statistical test. We have amended the Results sentence to specify the analysis used and to report the relevant statistics:

*“We conducted separate one-way ANOVAs at each of the six test frequencies (1–8 kHz) and found no significant group differences in DPOAE DP1 amplitudes (1 kHz: F (2, 75) = 1.68, p = 0.193; 2 kHz: F = 0.31, p = 0.732; 3 kHz: F = 0.86, p = 0.429; 4 kHz: F = 1.18, p = 0.312; 6 kHz: F = 0.39, p = 0.681; 8 kHz: F = 0.09, p = 0.919; see Figure 1A).”*

This revision explicitly states that one-way ANOVA was applied at each frequency and provides the corresponding F‐ and p-values.

**Specific Comment (Line 231):** ***“Here the ANOVA statistic is reported. Why was it not done in the previous cases?”***

**Response:** Thank you for drawing our attention to this inconsistency. You are correct that several earlier results were reported without the corresponding test statistics. We have re-examined all analyses and now provide the appropriate statistics—F- or *t*-values with their degrees of freedom and *p*-values—for every comparison (THI, VAS, TEOAE, GDT, MHINT, age, audiometry, etc.). The Results section and relevant table notes have been updated accordingly, ensuring that each finding is fully documented and presented in a uniform format. We appreciate the opportunity to improve the clarity and completeness of our statistical reporting.

**Specific Comment (Line 245):** ***“In which figure are the results of the GDT test shown? Figure 2? This is not referenced to in the text? The Y-axis label in figure should be changed to ‘******Gap detection threshold [ms]’. Currently it says ‘time’ which is misleading, as it actually depicts the GDT. Why do the sample sizes differ from the sample sizes described in the methods? 30/30/30 vs. 30/21/30?”***

**Response:** Thank you for these helpful suggestions. We have addressed each point as follows:

1. **Figure reference** – The GDT paragraph in the Results now begins:
   *“Figure 2 illustrates the gap-detection thresholds for the three groups. A two-way ANOVA (group × frequency) revealed …”*
2. **Axis label** – In Figure 2 the Y-axis has been relabelled **“Gap detection threshold (ms)”, and the caption has been updated accordingly.**
3. **Sample-size transparency** – Nine acute-tinnitus participants could not complete the full Gap diction test owing to time constraints or test fatigue. The final sample for this measure is therefore n = 21 (acute), 30 (chronic), 30 (control). These numbers now appear in the Figure 2 caption for the GDT.
4. **Methodological note added** – To explain such discrepancies throughout the manuscript, the Participants subsection now includes:

*“**Not every participant was able to complete every behavioral task because of time constraints or fatigue; therefore, the effective sample size (n) varies by test. The exact n for each analysis is provided in the corresponding figure or table legend.”*

These revisions clarify the figure citation, correct the axis label, and make our sample sizes fully transparent.

**Specific Comment (Line 291):** ***“Why should there be a difference in acute tinnitus and chronic tinnitus in the first place? This is not fully clear, and should be explained more clearly.”***

**Response:** Response: Thank you for this thoughtful comment. We have now clarified the rationale for comparing acute and chronic tinnitus in both the Introduction and Discussion. Specifically, we explain that while some models suggest chronic tinnitus may involve cumulative maladaptive plasticity or sustained emotional burden that exacerbates auditory dysfunction, it is also plausible that the main central changes occur early and subsequently stabilize. Our results support the latter view, showing that central auditory deficits are already present in the acute phase and do not worsen with chronicity—suggesting that partial adaptation may limit further deterioration. This distinction helps refine our understanding of tinnitus progression and underscores the importance of early intervention.

**Revised:**

*“While some studies suggest chronic tinnitus may involve cumulative maladaptive plasticity or sustained emotional burden that exacerbates auditory dysfunction,with greater neural changes observed in regions involved in emotional regulation, cognitive control, and multisensory integration—such as the hippocampus, amygdala, and frontal cortex—our findings support an alternative view: that early central changes emerge shortly after onset but remain relatively stable over time, likely due to partial neural adaptation.”*

**Specific Comment (Line 299):** ***“How is it linked to the multimodality network? On what research is this assumption based, please provide some references. Which non-auditory regions does it affect? Please elaborate with adequate references.”***

**Response:** Thank you for this valuable comment. We have revised the relevant section to clarify how tinnitus is linked to a multimodal brain network, and we now provide supporting references. Specifically, we highlight that tinnitus engages not only the auditory cortex but also non-auditory regions such as the amygdala, hippocampus, parahippocampal gyrus, anterior cingulate, precuneus, and dorsolateral prefrontal cortex (Chen et al., 2017; Lan et al., 2021; Singh et al., 2023). These areas are involved in emotion, memory, and attention, and their involvement helps explain why emotional distress in the acute stage correlates with impaired auditory performance. This revision has been incorporated into the Discussion section to address the reviewer’s concern.

Revised:

*“High distress in the acute stage is linked to poorer speech recognition performance, linking our data to neuro-imaging evidence that tinnitus engages a multimodal network encompassing auditory cortex and non-auditory hubs such as the amygdala, hippocampus, parahippocampal gyrus, anterior cingulate, precuneus, and dorsolateral prefrontal cortex (Chen et al., 2017; Lan et al., 2021; Singh et al., 2023). These regions mediate emotion, memory, and attention, helping to explain why heightened early distress magnifies listening difficulties while later limbic adaptation spares further sensory decline.”*

**Specific Comment (Line 410–412):** ***“This is an interpretation, but this is not shown in this study. Please tone this down, and point out that this is a hypothesis. This is a bit of an overinterpretation of the results. Although they show a relationship here, they do not show causal effects between the different variables. The assumption that the emotional management directly affects the central auditory system is a speculation.”***

**Response:** Thank you for pointing out the need to avoid causal language. We have re-worded the passage to present this idea as a hypothesis rather than a proven mechanism:

*“Although causality cannot be inferred from a cross-sectional design, these findings generate the testable hypothesis that* *effective emotional management might help disrupt the distress-auditory feedback loop and could prevent further functional decline.”*

This revised wording introduces the necessary qualifiers (“associated with”, “hypothesise”, “could”) and explicitly acknowledges that causality has not been established.

**Specific Comment (Line 420):** ***“It is not fully clear what is the novel aspect of this study, because it is already known that managing the psychological components of tinnitus is already known. What is the specific new contribution of the current study? This should be pointed out in more detail.”***

**Response:** Thank you for this insightful comment. We have revised the Discussion and Conclusion sections to better emphasize the novel contributions of our study, beyond the well-established role of psychological factors in tinnitus. Specifically:

- To our knowledge, this is the first study to behaviourally demonstrate that central-auditory deficits emerge within the first three months of tinnitus and remain stable over time, despite normal outer hair cell function. By directly comparing acute and chronic tinnitus groups with normal audiograms, we show that impairments in efferent suppression, temporal resolution, and speech-in-noise recognition are already present in the acute phase and do not worsen in chronic cases—suggesting early plateauing of central dysfunction, a finding not clearly established in prior research.
- Moreover, we provide quantitative evidence linking emotional distress to auditory performance during the acute phase. Specifically, THI emotional and catastrophic subscale scores were significantly correlated with speech-in-noise thresholds, indicating that heightened psychological burden may intensify the perceptual impact of tinnitus early in its course. This offers new insight into the temporal dynamics of emotion–auditory interaction in tinnitus.

These novel findings have now been clearly stated in the revised Discussion and Conclusion to highlight our study’s contributions.

**Revised:**

**“***This study is the first to demonstrate behaviourally that central-auditory deficits appear within the first three months of tinnitus and remain unchanged in the chronic stage, despite intact outer-hair-cell function. Adults with normal audiograms but bothersome tinnitus showed markedly reduced contralateral suppression of otoacoustic emissions, prolonged gap-detection thresholds, and poorer speech-in-noise perception—deficits of identical magnitude in both acute and chronic cohorts. High distress in the acute stage is linked to poorer speech recognition performance.***”**

**Specific Comment (Line 438):** ***“The limitations are not discussed in enough detail. How does the small sample size affect the generalisability? Why is the current sample not generalisable, for example due to age? What type of cohorts need to be included? Please elaborate.”***

**Response:** Thank you for asking us to elaborate on the study’s limitations and their impact on *generalisability*. We have expanded the Limitations section to address sample size, age range, hearing status, and study design, and we outline the cohorts and methods that future work should include. The revised paragraph now reads:

***“Limitations.*** *This single-centre study enrolled a modest sample (n = 30 per tinnitus group) of adults aged 18–55 years with normal audiograms; a larger, multi-site cohort spanning younger and older patients—including those with concomitant hearing loss—will be needed to confirm the present findings. Because the design was cross-sectional, we could not follow neuroplastic changes as tinnitus progresses; prospective longitudinal studies coupled with EEG or fMRI are required to chart that trajectory. Emotional distress was assessed only with THI and VAS; adding validated anxiety- and depression scales, and analysing data with multivariate or machine-learning methods, would provide a fuller account of the psychological factors that interact with auditory function.”*

We trust this fuller discussion meets the reviewer’s request.

**Specific Comment (Line 444):** ***“There seem to exist two conclusion section, as 410-418 also already constitutes a conclusion of the results. This is confusing. Combine the two paragraphs into one conclusion at the end of the paper.”***

**Response:** Thank you for noting the duplicate conclusion sections. We have merged the former “Significance” paragraph and the interim “In conclusion …” paragraph into one concise Conclusion placed at the very end of the manuscript—after the Limitations. All earlier, redundant concluding sentences have been deleted. The new single Conclusion now:

*“**In adults with normal hearing who experience tinnitus, we demonstrate that a stable central-auditory deficit—characterized by weakened efferent suppression, prolonged gap detection, and impaired speech-in-noise recognition—emerges soon after onset and does not deteriorate with chronicity. Its perceptual burden, however, varies with emotional load: high distress in the acute stage is linked to poorer auditory performance, whereas later emotional adaptation lessens this interference. Although causality cannot be inferred from a cross-sectional design, these findings generate the testable hypothesis that effective emotional management might help disrupt the distress-auditory feedback loop and could prevent further functional decline. Recognizing tinnitus as a multisystem condition underscores the need to incorporate psychological support into standard care pathways, aiming to alleviate distress and improve auditory-related quality of life.”*

We have addressed every comment from the reviewers and the Academic Editor. The manuscript has been thoroughly revised—clarifying the Introduction and Methods, adding missing methodological detail, tempering speculative statements, and meeting all formatting and data-reporting requirements. All amendments are tracked in the revised file. We believe these changes have substantially strengthened the paper and now bring it fully in line with PLOS ONE’s publication criteria.

Thank you again for the constructive feedback and for considering our resubmission. Because the revised article is needed for first author’s forthcoming degree application, I would be grateful for a decision at your earliest convenience.

**Sincerely,**
Zhiwu Huang
